# Evaluation of Serum Long Non-Coding RNAs HOXA Transcript at the Distal Tip (HOTTIP), Colon Cancer–Associated Transcript 1 (CCAT1), and Colon Cancer–Associated Transcript 2 (CCAT2) as Potential Biomarkers in Schizophrenia

**DOI:** 10.5152/eurasianjmed.2026.251271

**Published:** 2026-06-12

**Authors:** Esra Güzel Tanoğlu, Filiz Ekim Çevik, Kadriye Nur Çakmur, Muhammed Fevzi Esen, Fatma Rumeysa Uzun, Murat Erkıran

**Affiliations:** 1 Department of Molecular Biology and Genetics, Institution of Hamidiye Medical Sciences, University of Health Sciences, İstanbul, Türkiye; 2 Experimental Medicine Research and Application Center, University of Health Sciences, İstanbul, Türkiye; 3 Department of Medical Sciences, Institute of Forensic Sciences and Legal Medicine, İstanbul University-Cerrahpaşa, İstanbul, Türkiye; 4 Department of Psychiatry, Bakırköy Training and Research Hospital for Psychiatry, Neurology and Neurosurgery, University of Health Sciences, İstanbul, Türkiye; 5 Health Information System, University of Health Sciences, İstanbul, Türkiye

**Keywords:** Biomarker, HOTTIP, long non-coding RNA, schizophrenia

## Abstract

**Background:**

Schizophrenia (SZ) is a chronic neuropsychiatric disorder with marked impairments in cognitive, behavioral, and emotional functions. Despite extensive research, reliable molecular biomarkers of the disease have not yet been identified. Long non-coding RNAs (lncRNAs) stand out as regulatory molecules with diagnostic potential in many neurological and psychiatric disorders. This study aimed to evaluate the serum levels of HOXA transcript at the distal tip (HOTTIP), colon cancer–associated transcript 1 (CCAT1), and CCAT2 lncRNAs as potential biomarkers in SZ.

**Methods:**

Seventy antipsychotic-naive patients with SZ and 55 healthy controls participated in the study from June 2023 to May 2024. Control and SZ serum samples were collected, and the relative expression levels of HOTTIP, CCAT1, and CCAT2 were analyzed using reverse transcription-quantitative polymerase chain reaction.

**Results:**

Results showed that HOTTIP expression was significantly increased in patients with SZ (*P* < .05). However, no significant difference was observed in CCAT1 and CCAT2 levels between the groups. The association between increased HOTTIP levels and clinical symptom profiles suggests that this molecule may be linked to disease activity and SZ response. The HOTTIP showed modest discriminative ability (AUC = 0.60, 95% CI 0.50-0.70).

**Conclusion:**

: The study was powered to detect moderate group differences; the null results for CCAT1/CCAT2 therefore exclude moderate-or-larger effects but not small effects. HOTTIP may represent a noninvasive biological marker associated with SZ, although its standalone diagnostic utility appears limited.

**Main Points**

- HOXA transcript at the distal tip (HOTTIP) long non-coding RNA is significantly upregulated in the serum of antipsychotic-naive schizophrenia (SZ) patients compared to healthy controls.
- Elevated HOTTIP correlates with clinical symptom severity, implying a potential link to disease activity or treatment response.
- The HOTTIP exhibits discriminatory power in distinguishing SZ patients from healthy controls, indicating that it carries a biologically relevant signal associated with SZ.

## Introduction

Schizophrenia (SZ) is a chronic and often lifelong, widespread neuropsychiatric disorder characterized by cognitive, emotional, and behavioral abnormalities.^1^^,^^2^ Genetic predisposition, neurobiological factors, prenatal and perinatal complications, prenatal stress, substance abuse, and social problems are among the environmental elements that play significant roles in the development and progression of SZ.^3^^-^^5^ Although genetic and environmental factors are thought to contribute to the pathogenesis of SZ, its etiological mechanisms remain largely unclear.

Non-coding RNAs are proposed to play vital roles in human brain development and maturation, beginning in early pregnancy and continuing throughout life. This developmental influence may contribute to the evolution of advanced cognitive functions and self-awareness unique to humans.^6^ Long non-coding RNAs (lncRNAs) serve crucial regulatory roles in different biological functions and cellular processes such as metabolism, cell differentiation, and cell cycle control.^7^ Recent studies showed that lncRNAs are involved in the pathogenesis of many kinds of diseases.^8^ These molecules are now regarded as fundamental modulators of gene expression and function.^9^^,^^10^ Long non-coding RNAs are known as key post-transcriptional regulators of gene expression. Although their significance in brain development and neuroplasticity is established, recent studies suggest they also play regulatory roles in the pathogenesis of central nervous system disorders.^11^ The lncRNAs function at multiple levels of gene regulation, including nuclear organization, chromatin remodeling, transcriptional control, guidance of RNA-binding proteins, and modulation of mRNA stability.^12^^-^^14^ The impact of these molecules on neurodevelopmental processes and their association with dysfunctions in the nervous system have led to their consideration as potential biomarkers and therapeutic targets for SZ. Despite the identification of genetic mutations and aberrations in lncRNA expression and functionality associated with psychiatric disorders, the precise links between these alterations and the molecular pathophysiology of numerous neuropsychiatric diseases including SZ remain largely unelucidated.^15^^,^^16^

This study aimed to analyze the serum expression levels of the lncRNAs HOXA transcript at the distal tip (HOTTIP), colon cancer–associated transcript 1 (CCAT1), and CCAT2 in patients with SZ and to evaluate their potential association with the disease. The HOTTIP, CCAT1, and CCAT2 were chosen based on the well-characterized role of these non-coding RNAs in epigenetic regulation and chromatin-level regulation of gene expression patterns relevant to cellular differentiation processes. The primary rationale for selecting CCAT1, CCAT2, and HOTTIP in this study is their established capacity to modulate disruptions in synaptic plasticity, neuronal migration, and β-catenin-dependent gene regulation—processes critically implicated in the neurodevelopmental pathophysiology of SZ.^17^^-^^19^ There is also current evidence to suggest that the peripheral patterns of lncRNA gene expression may reflect the central neurobiological state through shared developmental patterns, regulatory circuits, and mechanisms of intercellular communication, such as the use of extracellular vesicles, rather than through a one-to-one transport process through the blood-brain barrier.^20^ Furthermore, the study examined whether these lncRNAs were potential biomarkers for SZ.

## Material and Methods

### Sample Collection

A total of 70 patients diagnosed with SZ and 55 healthy individuals were enrolled in the study from the Department of Psychiatry, Bakırköy Training and Research Hospital for Psychiatry, Neurology and Neurosurgery, University of Health Sciences. The diagnosis of SZ was established through clinical interviews conducted by independent expert psychiatrists in accordance with Diagnostic and Statistical Manual of Mental Disorders, 5th Edition criteria. The term antipsychotic-naive was defined as patients who had never received depot antipsychotics and had not used any oral antipsychotic medication for at least 2 weeks prior to blood sampling. Although this time frame is a protocol commonly accepted in the literature to minimize the acute effects of antipsychotic medications, it is acknowledged that this condition represents a short-term “drug-free period” rather than a lifetime absence of antipsychotic treatment. The patient group included individuals over the age of 18 who had not taken antipsychotic medication for more than 2 weeks and had no known chronic illnesses. Individuals diagnosed with other mental diseases, including major depressive disorder or obsessive-compulsive disorder, those presently on antipsychotic medication, and pregnant or breastfeeding women were excluded from the study. The control group consisted of healthy individuals over the age of 18 with no history of chronic illness, psychiatric disorders, or substance use. Accordingly, the control group was screened for the absence of psychiatric disorders by using the same structured clinical interview applied to the patient group, in order to exclude patients with subclinical psychosis risk. Basic metabolic parameters were recorded, such as body mass index and smoking status, to ensure group comparability, and individuals with known active inflammatory processes were excluded. Socio-demographic data such as gender, age, and marital status were collected from each participant. To assess the psychotic symptoms of patients with SZ, the Positive and Negative Syndrome Scale (PANSS) was employed. The PANSS evaluations were conducted following previously reported methodologies.^21^^,^^22^ Ethical approval was obtained from the Ethics Committee of University of Health Sciences (NO. 02.09.2022/20.09). Written informed consent was obtained from all participants who were briefed on the details of the study. This study was conducted in accordance with the Declaration of Helsinki (2013).^23^

### RNA Extraction and Quantitative Real-Time Polymerase Chain Reaction

Serum samples were collected from both healthy control groups and patients with SZ prior to treatment. Total RNA was isolated from serum using Trizol reagent (Invitrogen, CA, USA), as previously described. RNA concentrations were determined, and purity was checked using a NanoDrop (BioSpec) spectrophotometer to verify A260/A280 ratios between 1.8 and 2.055. The primer efficiencies of HOTTIP, CCAT1, CCAT2, and beta (β)-actin (ACTB( were verified by using standard curves (R^2^ > 0.99), and melt curve analysis for each run was carried out in order to verify single-product amplification with no primer dimers. Hemolysis was checked visually during serum separation, and samples with obvious discoloration were excluded to minimize contamination of cellular RNA. Complementary DNA (cDNA) synthesis was performed using a cDNA synthesis kit (ABM; USA). Reverse transcription-quantitative polymerase chain reaction (RT-qPCR) analysis was conducted using SYBR Master Mix (Invitrogen, CA, USA) with specific primers, including HOTTIP; F- 5’CCTAAAGCCACGCTTCTTTG’3, R-5’TGCAGGCTGGAGATCCTACT’3,^24^ CCAT1; F- GCAGGCAGAAAGCCGTATCT’3, R-5’ TCCCAGGTCCTAGTCTGCTT’3,^25^ CCAT2; F-5’ CCCTGGTCAAATTGCTTAACCT’3, R-5’TTATTCGTCCCTTTTTATGGAT’3.^26^ Amplification was conducted using a BioRad PCR system (Applied Biosystems, USA), with cycling parameters comprising an initial denaturation at 95°C for 10 minutes, succeeded by 40 cycles of 95°C for 15 seconds and 60°C for 60 seconds. A standard dissociation protocol was implemented to confirm that each amplicon represented a single product. All quantifications were standardized to ACTB expression levels. The RT-qPCR assays were conducted in triplicate for samples per group. Relative expression levels were calculated using the 2^−^^ΔΔCt^ method, with ACTB as the internal reference gene and the mean ΔCt value of the healthy control group used as the calibrator.

### Statistical Analysis

**Figure 1.**
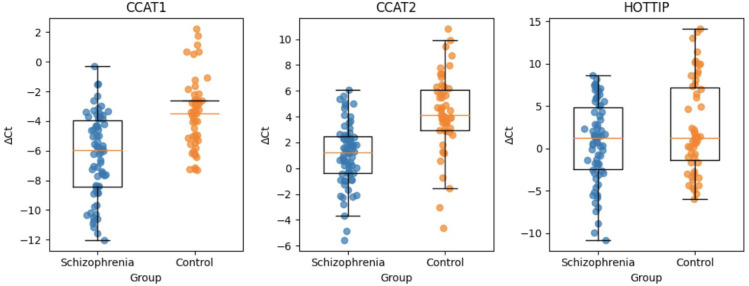
Distribution of serum lncRNA expression levels in schizophrenia and control groups. Boxplots show ΔCt distributions of (a) CCAT1, (b) CCAT2, and (c) HOTTIP in individuals with schizophrenia and healthy controls. Central line represents median, boxes indicate interquartile range (IQR), and whiskers extend to 1.5xQR. Given the non-normal distribution of the data, group comparisons were performed using the Mann–Whitney *U*-test.

A post-hoc sensitivity analysis was conducted to determine the minimal detectable effect size within the sample of 70 patients with SZ and 55 healthy controls (*α* = 0.05). This analysis showed that the study had 80% power to detect differences corresponding to a standardized mean difference of about *d *= 0.50, which is roughly equivalent to an AUC of 0.64. Descriptive statistics for each lncRNA were calculated. The Shapiro–Wilk test was employed to evaluate the normality of expression data, indicating non-normal distributions; therefore, ΔCt values were summarized using medians and interquartile ranges and visualized with boxplots (Figure 1). As a result, non-parametric statistical tests were performed. Pairwise comparisons of lncRNA expression levels between the SZ and control groups for each variable were conducted via Mann–Whitney *U*-test in SPSS 27.0 (IBM Corp., NY, USA). Statistical significance was defined at *P *< .05. Data were presented in graphical formats using GraphPad Prism 9 (San Diego, USA).

Missing data were handled using a complete-case approach. Participants with missing PANSS scores or demographic variables were excluded from analyses involving those variables, while all available data were retained for group-level comparisons. All lncRNA expression measurements were retained for group comparisons and ROC analyses. Participants with missing PANSS or demographic data (n = 6) were excluded only from analyses requiring those variables. Missing data were limited to clinical and demographic variables and were not associated with lncRNA measurement failure or study outcomes.

## Results

**Figure 2.**
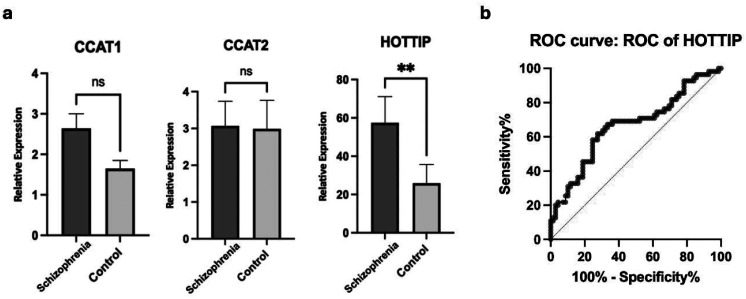
(a) Expression levels of CCAT1, CCAT2, and HOTTIP between SZ and control groups. (b) ROC curve for HOTTIP distinguishing SZ and controls.

Colon cancer–associated transcript 1 expression levels were comparable between the SZ group (n = 70) and control group (n = 55). The median expression in the SZ group was 1.34, while the control group exhibited a median of 1.41. The mean expression values (SZ: 2.64 ± 0.361 SEM; control: 1.65 ± 0.200 SEM) and overlapping 95% CIs (SZ group: 1.92-3.36; control: 1.25-2.05) suggested no significant disparity (Figure 1). Mann–Whitney *U*-test showed there was no difference between the groups (*P *= .714) (Figure 2a). Similar to CCAT1, CCAT2 expression showed no significant differences between SZ groups (n = 70; median = 1.02) and control groups (n = 55; median = 1.20). The mean expression values were 3.07 (±0.668 SEM) and 2.99 (±0.773 SEM) for SZ and control groups, respectively. The 95% CIs (SZ: 1.74-4.40; control: 1.44-4.54) overlapped substantially, and Mann–Whitney *U*-test corroborated the absence of statistical significance (*P *= .671) (Figure 2a). In contrast, HOTTIP expression was significantly elevated in the SZ group (n = 69; median = 5.34) compared to the control group (n = 55; median = 0.769) (Table 1). The SZ group exhibited a mean expression of 57.4 (±13.7 SEM), nearly double the control group’s mean (25.9 ± 9.82 SEM). Non-overlapping 95% CIs (SZ: 30.1-84.7; control: 6.20-45.6) and the test (*P *< .001) confirmed this difference (Figure 2a). The distribution of expression data underscores the non-normal nature of the dataset. While CCAT1 and CCAT2 showed similar expression levels across groups, HOTTIP demonstrated a pronounced SZ-associated upregulation. The study initially enrolled 70 participants; however, PANSS scores and demographic data were unavailable for 6 individuals. Final cohort included 64 participants with complete data (Table 2). Males constituted 67.2% (n = 43) and females 32.8% (n = 21). Educational attainment was distributed across elementary (28.1%; n = 18), secondary (29.7%; n = 19), high school (28.1%; n = 18), and university (14.1%; n = 9). 78.1% of people (n = 48) were single, 15.6% (n = 10) married, and 9.4% (n = 6) divorced. Descriptive statistics for age, HOTTIP expression, and PANSS scores are summarized in Table 3. Mann–Whitney test exhibited a significant gender difference in HOTTIP expression (*P *= .015), with males exhibiting higher mean expression (69.49 ± 14.89 SEM) compared to females (31.79 ± 20.16 SEM). No significant differences in HOTTIP expression were observed across literacy levels (*P *= .897) or marital status (*P *= 0.063). Spearman’s correlation analysis (Table 1) identified significant association among variables. The PANSS-N correlated with HOTTIP expression (*r *= 0.246, *P* = .047). However, HOTTIP expression showed no significant correlation with age (*r *= −0.092, *P *= .467), PANSS-G (*r *= 0.132, *P* = .298), PANSS-P (*r *= 0.117, *P* = .359), or total PANSS (*r *= 0.200, *P* = .114). In addition, PANSS total scores showed strong correlations with all subscales (PANSS-G: *r* = 0.898, *P* < .001; PANSS-P: *r *= 0.708, *P* < .001; PANSS-N: *r *= .723, *P* < .001). The ROC analysis for HOTTIP yielded AUC = 0.60 (95% CI 0.50-0.70; *P* = .002) with sensitivity 61.8% and specificity 72.5% for distinguishing SZ from controls (Figure 2a). This suggests that while a biological signal exists, the effect size is small and falls below the threshold typically required for reliable clinical screening. Sensitivity analysis indicated the study had 80% power to detect *d *= 0.50 (AUC = 0.64); observed effects for CCAT1/CCAT2 were tiny (AUC = 0.48), explaining the non-significant results (Figure 2b).

## Discussion

Schizophrenia is a complex psychiatric disorder defined by impairments in thought, perception, emotion, and behavior.^27^ Although several lncRNAs have been previously associated with SZ,^28^ ongoing biomarker studies continue to explore novel lncRNAs due to the incomplete mapping of lncRNA involvement in this disorder. For example, DISC2, which interacts with DISC1, has been reported to regulate neuronal development and synaptic plasticity.^29^ Other prominent lncRNAs implicated in SZ pathogenesis include NEAT1 and MALAT1, which are known to play critical roles in fundamental neurobiological processes like synaptic remodeling and dopaminergic signaling modulation.^30^^,^^31^ Particularly, lncRNAs detectable in cerebrospinal fluid have demonstrated high potential as non-invasive biomarkers for clinical applications.

Colon cancer-associated transcript 1 has been implicated in processes like cell proliferation, migration, and invasion in multiple cancer types, including liver, gallbladder, prostate, and colorectal cancers.^32^ Although this study represents the first comprehensive investigation comparing the serum expression profiles of CCAT1 and CCAT2 in patients with SZ and healthy controls, no differences were found between the 2 groups. In contrast, another examined lncRNA, HOTTIP has previously been reported to be upregulated in various cancers including breast, gynecological, gastrointestinal, pancreatic, colorectal, prostate, lung, and glioma tumors.^33^^-^^35^ In the present study, HOTTIP expression was markedly increased in SZ patients relative to healthy controls. The most striking finding was the clear sex-related difference in HOTTIP expression: higher levels in male patients along with a significant correlation with the severity of negative symptoms.

Although the observed upregulation of HOTTIP suggests a possible involvement in SZ-related biological processes, the modest effect sizes and limited discriminative performance indicate that these findings should be interpreted as preliminary and hypothesis-generating rather than confirmatory. Current literature indicates that HOTTIP orchestrates the HOXA gene cluster^20^ during neuronal differentiation, modulates pathways related to synaptic plasticity,^36^ and plays critical roles in controlling gene expression via epigenetic mechanisms. These pioneering results underscore the need for further mechanistic studies and validation in larger cohorts to clarify HOTTIP’s role in SZ.

Although HOTTIP expression differed significantly between patients with SZ and controls, its discriminative performance was modest (AUC = 0.60). This level of accuracy indicates limited standalone diagnostic utility and suggests that HOTTIP alone is insufficient for reliable case-control classification. Rather than serving as a diagnostic biomarker, elevated HOTTIP levels may reflect underlying disease-related biological processes, particularly those associated with negative symptom severity, as suggested by its correlation with PANSS-N scores. However, the observed correlations were weak in magnitude (*r *= 0.20-0.25) and, although statistically significant, may have limited clinical relevance.

To the best of knowledge, this is the first study showing expression changes of CCAT1, CCAT2, and HOTTIP lncRNAs in the serum of patients with SZ, proposing these molecules as potential biological markers. Nevertheless, it is critical to emphasize that the observed upregulation of HOTTIP reflects an underlying biological process rather than a clinically actionable diagnostic tool at this stage. Moreover, it distinguishes itself as the first study assessing lncRNA expression in SZ using non-invasive serum samples, offering both tissue specificity and practical applicability for biomarker screening in clinical settings.

This study has several limitations. Its single-center design limits the external validity and generalizability of the findings to broader and more diverse SZ populations. Sample size was limited, and the single-center design may constrain the applicability of the results. Patient characteristics, clinical profiles, and healthcare practices at a single institution may not fully represent those of other settings. Moreover, the lack of external validation in an independent cohort means the results should be interpreted with caution. Additionally, comorbid substance use was assessed through clinical interviews and medical record reviews rather than systematic urine toxicology screening, which may not fully exclude undisclosed substance use. In particular, the negative findings for CCAT1 and CCAT2 likely rule out moderate or large effects but remain compatible with small effects that could not be detected with the current sample. In light of these challenges, future studies need to utilize longitudinal approaches in order to see whether there is variation in the expression of HOTTIP with antipsychotic therapy that would help distinguish between state and trait markers. Moreover, there is a need for further analyses that will examine whether there is a direct modulation of dopaminergic or glutamatergic pathways in SZ models. Future research may aim to replicate these results in independent populations and explore whether combining HOTTIP with other lncRNAs or clinical markers may improve discriminative performance beyond that of HOTTIP alone. Additionally, in psychiatric disorders characterized by substantial clinical and molecular heterogeneity, modest AUC values are common in early candidate biomarker discovery studies. In this context, the observed AUC may be interpreted as evidence of biological signal rather than clinical discriminative power. Future studies with much larger cohorts are required to determine if HOTTIP could provide value only when integrated into a larger panel of biomarkers.

Accumulating knowledge of lncRNAs has provided new insights into the molecular mechanisms underlying SZ and the development of practical, sensitive diagnostic and therapeutic tools. However, further studies are needed to understand the mechanisms of lncRNA dysregulation and their targets in the development of SZ. Furthermore, diagnosing SZ using biomarkers is crucial for preventing progression in patients; however, sensitive and effective biomarkers remain unidentified. This study found that HOTTIP may represent a promising biological marker associated with SZ pathophysiology, although its standalone diagnostic performance appears limited.

## Figures and Tables

**Table 1.** Spearman’s Correlation Matrix for HOTTIP, Age, and PANSS Subscales

		**HOTTIP**	**Age**	**PANSS (G)**	**PANSS (P)**	**PANSS (N)**	**PANSS (TOTAL)**
HOTTIP	*r*	–					
	N	64					
Age	*r *	−0.092	–				
	*P *	.467					
	N	64	64				
PANSS (G)	*r *	0.132	0.082	–			
	*P *	.298	.519				
	N	64	64	64			
PANSS (P)	*r *	0.117	0.312^*^	0.510^**^	–		
	*P *	.359	.012	.000			
	N	64	64	64	64		
PANSS (N)	*r *	0.246^*^	0.094	0.528^**^	0.199	–	
	*P *	.047	.459	.000	.115		
	N	64	64	64	64	64	
TotalPANSS	*r *	0.200	0.187	0.898^**^	0.708^**^	0.723^**^	–
	*P *	.114	.139	.000	.000	.000	
	N	64	64	64	64	64	64

^*^Correlation is significant at the 0.05 level (2-tailed).

^*^Correlation is significant at the 0.01 level (2-tailed).

**Table 2.** Demographic Characteristics Stratified by Gender

**Variables**		**Gender**				**Total**
		**Male**		**Female**		
		**n**	**%**	**n**	**%**	
Literacy	Elementary	11	25.6	7	33.3	18
	Secondary	16	37.2	3	14.3	19
	High school	12	27.9	6	28.6	18
	University	4	9.3	5	23.8	9
Marital Status	Married	2	4.7	8	38.1	10
	Single	39	90.7	9	42.9	48
	Divorced	2	4.7	4	19	6

**Table 3.** Descriptive Statistics for Age, HOTTIP Expression, and PANSS Scores

**Variables**	**Mean**	**S.E.**	**Max.**	**Min.**	**Median**
Age	40.34	1.52	63.00	18.00	39.00
HOTTIP	57.12	12.10	409.54	.02	8.51
PANSS (G)	64.78	1.16	92.00	47.00	64.50
PANSS (P)	35.39	.82	50.00	17.00	35.00
PANSS (N)	31.56	.84	47.00	18.00	32.00
Total PANSS	131.63	2.21	167.00	87.00	134.00
